# Effects of Exercise Training on Bone Health Parameters in Individuals With Obesity: A Systematic Review and Meta-Analysis

**DOI:** 10.3389/fphys.2021.807110

**Published:** 2022-02-14

**Authors:** Hassane Zouhal, Abdel Jalil Berro, Sarah Kazwini, Ayoub Saeidi, Ayyappan Jayavel, Cain C. T. Clark, Anthony C. Hackney, Trisha A. VanDusseldorp, Abderraouf Ben Abderrahman, Rawad El Hage

**Affiliations:** ^1^University Rennes, M2S (Laboratoire Mouvement, Sport, Santé), Rennes, France; ^2^Institut International des Sciences du Sport (2I2S), Irodouer, France; ^3^Division of Education, Faculty of Arts and Sciences, Department of Physical Education, University of Balamand, Tripoli, Lebanon; ^4^Department of Physical Education and Sport Sciences, Faculty of Humanities and Social Sciences, University of Kurdistan, Sanandaj, Iran; ^5^SRM College of Physiotherapy, SRM Institute of Science and Technology, Chennai, India; ^6^Centre for Intelligent Healthcare, Coventry University, Coventry, United Kingdom; ^7^Department of Exercise & Sport Science, University of North Carolina, Chapel Hill, NC, United States; ^8^Department of Nutrition, University of North Carolina, Chapel Hill, NC, United States; ^9^Department of Exercise & Sport Management, Kennesaw State University, Kennesaw, GA, United States; ^10^Higher Institute of Sport and Physical Education of Ksar-Said, University of Manouba, Tunis, Tunisia

**Keywords:** bone health, exercise, bone mineral density, bone mineral content, resistance exercise and aerobic exercise, combined training

## Abstract

**Background:**

Osteoporosis causes bone fragility, increasing the risk of fractures. Evidence suggests a strong correlation between obesity and fracture risk. Physical training is known to enhance bone resistance and protect from fracture; however, its osteogenic effect in the presence of obesity remains unknown.

**Objective:**

We sought to evaluate the influence of exercise training on bone health indices in individuals with obesity.

**Methods:**

This systematic literature search was conducted using common electronic databases from inception - December 2019. The following key terms (and synonyms searched for by the MeSH database) were included and combined using the operators “AND,” “OR,” “NOT”: [(“body mass index” OR obesity OR obese OR overweight OR fat mass) AND (“bone mineral density” OR “bone mineral content” OR “peak bone mass” OR “mechanical loading” OR “Osteoporosis” OR “bone geometry” OR “bone resistance”) AND (“exercise training” OR “physical training” OR “strength training,” OR “resistance training” OR “aerobic training” OR “combined training”)].

**Results:**

After screening, 10 studies (889 initial records) were included in the final analysis (8 different countries, 263 participants). Two studies investigated males, six females, and two, both sexes. The training duration was at least eight weeks with 2–3 sessions/week. Physical training displayed a significant trivial impact on the whole body (WB) BMD (0.13 SMD; 95% CI [0.00, 0.26], *p* = 0.046). Subgroup analyses indicated a significant small increase in the WB BMD (0.27 SMD; 95% CI [0.00, 0.53], *p* = 0.048) in the endurance training group, a non-significant trivial increase in the WB BMD (0.11 SMD; 95% CI [−0.06, 0.29], *p* = 0.203) in the resistance group, and a non-significant trivial increase in the WB BMD (0.03 SMD; 95% CI [−0.26, 0.32], *p* = 0.86) in the combined training group. In addition, a significant small decrease was found in the weight of trained subjects (−0.24 SMD; 95% CI [−0.42, −0.05], *p* = 0.011).

**Conclusion:**

Physical training has little to no effect on the WB BMD in subjects with overweight/obesity. Currently, insufficient evidence to advocate for any specific type of exercise for enhancing bone health exists for overweight/obese individuals. Investigations examining the impact of varying types of physical exercise on WB BMD of obese individuals are needed.

## Introduction

The worldwide incidence of obesity continues to grow and is largely attributable to an imbalance between energy intake and energy expenditure, which leads to an increase in body fat accumulation, which adversely affects health (Gonnelli et al., 2014). For example, the accumulation of adipose tissue is associated with various non-communicable disorders, including cardiovascular disease, type 2 diabetes, and cancers (Cao, 2011). Moreover, recent data has demonstrated a pathophysiological link between adiposity and osteoporosis (Cortet and Roux, 2016), where it is suggested that there is a decreased bone mineral density (BMD) and an increase in the fracture risk among obese subjects (Shapses and Sukumar, 2012; Gonnelli et al., 2014; Cortet and Roux, 2016).

Osteoporosis is a systemic skeletal disease characterized by a low BMD and microarchitectural deterioration of bone tissue, which leads to an increased risk of developing spontaneous and traumatic bone fractures (Health, 2001), and it represents a major contributor to the global burden of disease (Torres-Costoso et al., 2020). Genetic and hormonal factors, poor diet, excess caloric intake, and/or lower physical activity are known to be involved in the development of obesity and osteoporosis (Shapses and Sukumar, 2012; Weaver et al., 2016).

The relationship between adipose and bone tissue is complex; indeed, a greater body-weight is generally considered to be beneficial to bone health due to the positive effect of mechanical loading on bone formation (Cao, 2011). Moreover, a decreased body mass index (BMI) is a risk factor for lower BMD and predicts greater bone loss in older individuals (Nguyen et al., 1998). In contrast, the potentially positive influence of fat accumulation (i.e., increased weight) on bone health remains controversial; indeed, evidence suggests that excessive adiposity may be detrimental to bone health and increases fracture risk (Shapses and Sukumar, 2012). Furthermore, studies have suggested that the associated increases of BMD and weight are not proportional. Researchers have found in individuals with obesity, that composite indices of femoral neck strength and BMD per unit BMI are significantly lower compared to individuals with normal-weight (Laet et al., 2005; El Khoury et al., 2017). Also, several authors have indicated that fat distribution affects the bone differently; while subcutaneous fat is positively associated with bone mass, visceral and marrow fat are negatively correlated with BMD (Gilsanz et al., 2009; Russell et al., 2010).

Obesity putatively affects bone metabolism through several mechanisms. Both osteoblasts and adipocytes are derived from a common mesenchymal stem cell and agents inhibiting osteoblastogenesis and increasing adipogenesis (Cao, 2011). Obesity is also associated with chronic inflammation, and adipocytes secrete proinflammatory cytokines which can promote osteoclast activity and bone resorption (Cao et al., 2005). In addition, it has been demonstrated that obesity and osteoporosis may be associated with increased production of proinflammatory cytokines and elevated oxidative stress (Wellen and Hotamisligil, 2003; Mundy, 2007). The excessive secretion of leptin and/or decreased production of adiponectin by adipocytes in obesity may also either directly affect the bone formation or indirectly affect bone resorption (Elefteriou et al., 2004; Oshima et al., 2005). Finally, a high-fat diet may alternate intestinal calcium absorption resulting in a decrease in calcium availability for bone formation (Nelson et al., 1998).

Due to the increased prevalence of osteoporosis, non-pharmacological treatment and/or preventive strategies are highly sought-after. Epidemiological and clinical trial research confirms the positive impact of regular physical activity on bone and body composition (Slemenda et al., 1991; Bailey et al., 1999; Specker and Binkley, 2003), and physical exercise is capable of maximizing peak bone mass in younger years and minimizing age-related bone loss in individuals with lean (Babatunde and Forsyth, 2013). These effects are dependent, primarily, on the type of exercise; high impact and resistance exercises are the most recommended strategies to increase BMD and thereby increase bone resistance and decrease fracture risk (Kelley et al., 2001; Weaver et al., 2016). The majority of published studies have investigated the influence of this type of training and showed positive, consistent results (Guadalupe-Grau et al., 2009). Conversely, relatively fewer studies have explored in individuals with obesity the impact of aerobic and combined physical activities (PA) on bone parameters, and incumbent findings remain controversial (El Hage et al., 2009; Guadalupe-Grau et al., 2009). PA modulate bone remodeling through mechanical stimuli, which results in improvements in mineralization and bone geometry. In addition, PA can optimize body composition by increasing lean mass and decreasing fat mass (Guadalupe-Grau et al., 2009).

How obesity influences the effect of PA on bone health is, so far, unclear (Menzel et al., 2015), and the generalizability of previous research conducted in subjects with normal-weight to individuals with overweight/obesity is ambiguous. To date, aerobic, resistance, and other types of exercises have been used to explore the osteogenic response in the presence of obesity, although diverse results have been recorded. Therefore, the first aim of this systematic review and meta-analysis was to assess the impact of PA on bone health indices in individuals with obesity and the second to investigate whether the type of PA can modulate this effect.

## Methods

### Eligibility Criteria

PICOS (Population, Intervention, Comparison, Outcome, and Study design) criteria were used as the inclusion criteria for the current review (see Table 1) (Moher et al., 2015). This systematic review included original studies (randomized or non-randomized) for which the full texts were available and that performed interventions with exercise training, included two or more weeks of follow-up, involved individuals with overweight or obesity (BMI ≥ 25 kg.m^−2^), included one or both sexes, and specifically evaluated whole-body BMD, before and after the intervention, in individuals with obesity.

**Table 1:** PICOS (participants, interventions, comparisons, outcomes, study design).

**PICOS component**	**Detail**
Participants	Individuals with overweight or obesity (BMI ≥ 25 kg.m^−2^; body fat > 25% for men and >30% for women)
Interventions	Exercise training two or more weeks of follow-up (aerobic, resistance and combined training)
Comparisons	Control group/Untrained participants
Outcomes	Physical performances, bone mineral density, bone mineral content, bone geometry, hormone responses.
Study designs	nRCTs, nRnCTs and RCTs

*nRCT, non-randomized controlled trial; nRnCT,non-randomized non-controlled trial; RCT, randomized controlled trial*.

Studies were excluded if they **(1)** did not meet the minimum requirements of an experimental study design (e.g., case reports), **(2)** did not meet the minimum requirements regarding training design (e.g., lack of information on volume, frequency, training methodology), **(3)** were not written in English or French, **(4)** involved individuals were not overweight/obese, **(5)** did not include the measurement of whole body bone mineral density (WB BMD) or **(6)** did not include sufficient information that allows the determination of effect size. Additionally, the following exclusion criteria were adopted to reduce confounding factors: duplicate publications or sub-studies of included studies, studies involving pathologies, and studies associating exercise with a nutritional intervention (e.g., nutrition counseling, balanced or hypocaloric diets, and supplements) or pharmacological drugs. Moreover, review articles were not included in the current systematic review.

### Literature Search Strategy

This systematic review and meta-analysis is reported in accordance with the Preferred Reporting Items for Systematic Reviews and Meta-Analyses (PRISMA) statement and the Cochrane Handbook for Systematic Reviews of Interventions (Higgins and Deeks, 2011).

We searched the following electronic databases (up to December 2019) without a period limit: Cochrane Library, PubMed, Science Direct, Scopus, SPORTDiscus, and Web of Science. Additionally, a manual search for published studies in Google Scholar was conducted for the gray literature analysis. The following key terms (and synonyms searched for by the MeSH database) were included and combined using the operators “AND,” “OR,” “NOT”: [(“body mass index” OR obesity OR obese OR overweight OR fat mass) AND (“bone mineral density” OR “bone mineral content” OR “peak bone mass” OR “mechanical loading” OR “Osteoporosis” OR “bone geometry” OR “bone resistance”) AND (“exercise training” OR “physical training” OR OR “strength training,” OR “resistance training” OR “aerobic training” OR “combined training”)]. Only eligible full texts in English or French were considered for analysis.

Three investigators among the authors (AB, KS, and REH) independently performed searches in the electronic databases, and disagreements were solved by consensus.

### Study Selection

**Figure 1:**
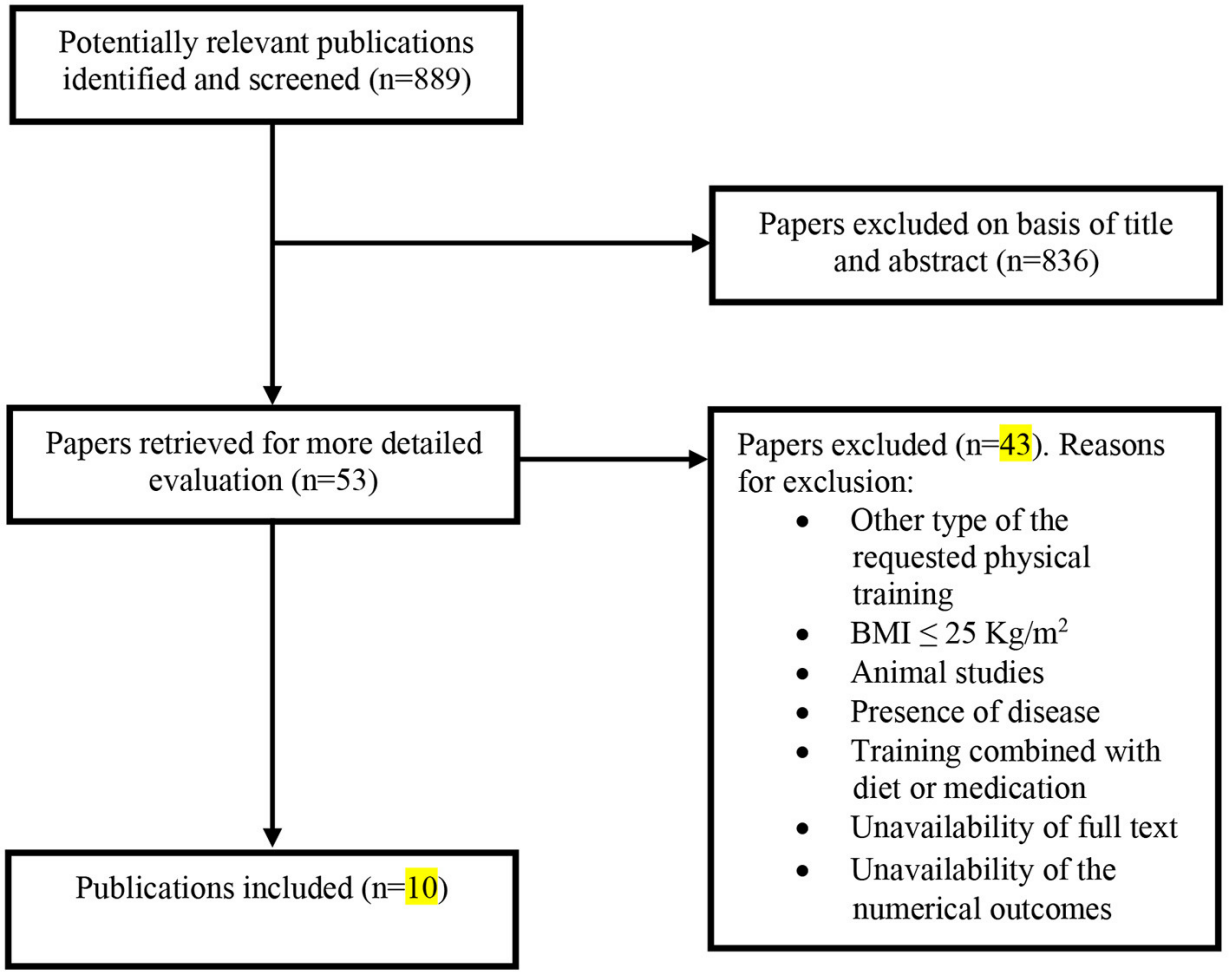
Flow diagram for the selection of studies. Selection process for research articles (n = 10) included in this systematic review. Adapted version of the recommendations in the PRISMA (Preferred Reporting Items for Systematic Reviews and Meta-Analyses) statement (Moher et al., 2010).

Inclusion or exclusion of articles was decided with the application of the PICOS criteria (see Table 1) to the title, abstract, and/or full text of articles. The data-collection process is presented in Figure 1 (Liberati et al., 2009). The titles and abstracts of the selected articles were independently assessed by two researchers (AB and AJ). The reviewers were not blinded to the authors, institutions, or journals associated with the manuscripts. Abstracts that provided insufficient information about the inclusion and exclusion criteria were retrieved for full-text analysis. Furthermore, the researchers independently analyzed the full text and determined the eligibility of the studies, and disagreements were resolved by consensus. To avoid double-counting participants or to clarify questions about the methods, the corresponding authors were contacted if needed. Furthermore, when necessary, the corresponding author was also contacted *via* e-mail to provide data not included in the published research. Two researchers (AB and KS) independently performed the data extraction, and disagreements were resolved by consensus.

### Quality Assessment

Study quality was assessed using the Physiotherapy Evidence Database (PEDro) scale (http://www.pedro.fhs.usyd.edu.au), which has been shown to have good reliability and validity (Maher et al., 2003). The PEDro scale has 11 possible points that examine external validity (criterion 1) and internal validity (criteria 2–9) of controlled trials and whether there is sufficient statistical information for interpreting results (criteria 10–11). The items of the scale are: (i) eligibility criteria were specified; (ii) subjects were randomly allocated to groups; (iii) allocation was concealed; (iv) groups were similar at baseline; (v) subjects were blinded; (vi) therapists who administered the treatment were blinded; (vii) assessors were blinded; (viii) measures of key outcomes were obtained from more than 85 % of subjects; (ix) data were analyzed by intention to treat; (x) statistical comparisons between groups were conducted, and (xi) point measures and measures of variability were provided. The first criterion is not included in the final score. Moreover, because of the nature of physical activity interventions, patient and therapy blinding and allocation are difficult; therefore, the total score a trial could receive was 8 points. A cut-off of 6 on the PEDro scale was used to indicate high-quality studies, as this has been reported to be sufficient to determine high quality vs. low quality in previous studies (Maher et al., 2003). The studies were evaluated by two experienced investigators (AB and AJ) and in the event of disagreement, a third reviewer (HZ) was invited to review.

### Statistics

Effect sizes (ES) were computed to discern the standardized effect of acute and long-term training on the outcome variables (WB BMD (g/cm^2^) and weight (kg)). Mean group differences in WB BMD and weight between pre-and post-intervention periods were divided by a pooled SD, based on the assumption of a large correlation (*r* = 0.7) for each group. The standardized mean differences (SMD) were calculated to determine Cohen's *d* for each study and Hedge's g was used to account for potential bias in small sample sizes.

Given that BMD and weight values are continuous data, the weighted mean difference method was used for combining study effect size estimates. Weighting assigned to each study group comparison in the analysis was in the inverse proportion to the variance.

When data could be pooled, these effect sizes and 95% confidence intervals (95% CI) were calculated using random-effects models (DerSimonian and Laird approach), that account for true variation in effects between studies, as well as random error within a single study. Values for ES were defined as trivial (<0.2), small (0.2–0.6), moderate (0.6–1.2), large (1.2–2.0), and very large >2 (Hopkins et al., 2009). A negative effect size value indicated that the WB BMD or the weight decreased after the intervention, while a positive effect size indicated there was an increase. Cochrane's *Q* and the *I*^2^ index were used to assess heterogeneity. The alpha value for statistical significance for Q was set at *p* < 0.10, because it tends to suffer from low differential power (Higgins et al., 2003). For *I*^2^, values of 25, 50, and 75% were used to indicate low, moderate, and high heterogeneity, respectively (Higgins et al., 2003). Furthermore, *Z* test analysis was used to examine if the overall ES's were significantly different from zero.

We contacted authors to obtain any missing numerical data needed for analysis. For interventions with 2 or more arms including the same type of training (El Hage et al., 2009), in the meta-analysis, we combined the 2 arms using standard procedures, as outlined in Section 9.3.9 of the Cochrane Handbook (Higgins and Deeks, 2011). Subgroup analysis was used to investigate differences in the magnitude of the exercise-induced osteogenic effect across studies dependent to the type of training (endurance, resistance, and combined training). Potential publication bias was assessed through visual inspection of funnel plots. All statistical analyses were conducted using R (version 4.0.3; The R Foundation for Statistical Computing, Vienna, Austria) (The R Development Core Team; R Core Team, 2020) and the “metafor” package (Viechtbauer, 2010). Unless otherwise stated, *p* < 0.05 was used to demarcate statistical significance.

## Results

### Study Selection

Overall, our search yielded 889 records (Figure 1). After the screening of titles, abstracts, and full texts, 10 studies were included in our final analysis, and the characteristics of these studies are shown in Table 2. These studies were performed in 8 different countries (Lebanon, Canada, Brazil, USA, Taiwan, Korea, Spain, and Sweden), where 2 studies investigated only male subjects, 6 studies investigated only females, and 2 studies investigated both sexes. These papers included 15 training groups (9 for females, 5 for men, and 1 for men and females) and involved 263 trained participants in total. One study (Bocalini et al., 2009) included the measurement of body weight only. The training duration was at least 8 weeks and ranged, for the majority of studies, between 12 and 48 weeks. One study reported a training duration of 96 weeks (Warren and Chua, 2008). The training frequency was at least 2–3 sessions per week, and six studies reported compliance to the training, which ranged between 71 and 94%. The included long-term studies (24 > weeks) were classified as ‘high-quality’ studies (mean 8.2 in the PEDro scale score) (Table 3).

**Table 2:** Characteristics of the studies that examined the effect of exercise training on bone health indices in individual with obesity.

**Study**	**PEDro scale**	**Population/sex**	**Sample size**	**Country**	**Age, years (mean ±SD or range)**	**Characteristics of exercise training**	**Duration (weeks)**
**Endurance training**							
El Hage et al. (2009)	8	Women	N: 21 Ex1: normal weight: 7 Ex2: overweight: 8 Ex3: obese: 6	Lebanon	16.2 ± 1.8	Endurance training: running and collective game	12
Kim et al. (2015)	7	Men	N: 39 Ex: 29 CG: 10	Korea	25.3 ± 2.8	Aerobic exercise: treadmill running	8
Berro et al. (2020)	7	Women	N: 28 Ex: 14 CG: 14	Leba-non	18–35	Endurance training: treadmill running	48
**Resistance or Strength training**							
Huang et al. (2017)	9	Women	N: 35 Ex: 18 CG: 17	Taiwan	68.9 ± 4.9	Elastic band resistance training	12
Cunha et al. (2018)	10	Women	N: 62 Ex1S: 21 Ex3S: 20 CG: 21	Brazil	68.0 ± 4.3	Free weight and machines	12
Warren and Schmitz (2009)	9	Women	N: 148 Ex: 72 CG: 76	USA	36.4 ± 5.5	Strength training	96
Cornish and Chilibeck (2009)	8	Women and men	N: 51 ALA: 25 ALA men: 14 ALA women: 11 Pla: 26 Pla men: 14 Pla women: 12	Canada	65.4 ± 0.8	Resistance training	12
Romero-Arenas et al. (2013)	7	Women and men	N: 37 HRC: 16 ST: 14 CG: 7	Spain	61.6 ± 5.3	High-resistance circuit (HRC) training vs. traditional strength training (ST)	12
Bocalini et al. (2009)	7	Women	N: 25 Ex: 15 CG: 10	Brazil	57–75	Strength training	24
**Combined training**							
Bolam et al. (2016)	10	Men	N: 42 HI: 13 Mod: 15 CG: 14	Sweden	50–74	Upper body RE + high-dose impact loading or moderate dose impact loading	36
Choquette et al. (2011)	9	Women	N: 100 -Pla: 26 -ISO: 26 -Ex + Pla: 25 -Ex + ISO: 23	Canada	50–70	Resistance and aerobic exercise	24

*PEDro scale, physiotherapy evidence database scale; SD, standard deviation; N, number of subjects; G, group; C, control; Ex, exercise; RE, resistance exercise; HI, high-dose impact loading; MOD, moderate dose impact loading; Alpha-linolenic acid, ALA; ISO, isoflavones; Pla, Placebo*.

**Table 3:** Physiotherapy evidence database (PEDro) score of the included longitudinal studies.

**Study**	**Eligibility criteria**	**Randomized allocation**	**Blinded allocation**	**Group homogeneity**	**Blinded subjects**	**Blinded therapists**	**Blinded assessor**	**Drop out /15 %**	**Intention to treat analysis**	**Between-group comparison**	**Point estimates and variability**	**PEDro score**
El Hage et al. (2009)	•	•	◦	•	◦	◦	•	•	•	•	•	8
Kim et al. (2015)	•	•	◦	•	◦	◦	•	•	◦	•	•	7
Huang et al. (2017)	•	•	•	•	•	◦	•	•	◦	•	•	9
Cunha et al. (2018)	•	•	•	•	•	•	•	•	◦	•	•	10
Cornish and Chilibeck (2009)	•	•	•	•	◦	◦	•	•	◦	•	•	8
Romero-Arenas et al. (2013)	•	•	◦	•	◦	◦	•	•	◦	•	•	7
Bocalini et al. (2009)	•	•	◦	•	◦	◦	•	•	◦	•	•	7
Bolam et al. (2016)	•	•	•	•	•	◦	•	•	•	•	•	10
Choquette et al. (2011)	•	•	•	•	•	◦	•	•	◦	•	•	9
Berro et al. (2020)	•	•	◦	•	◦	◦	•	•	◦	•	•	7

**Figure 2:**
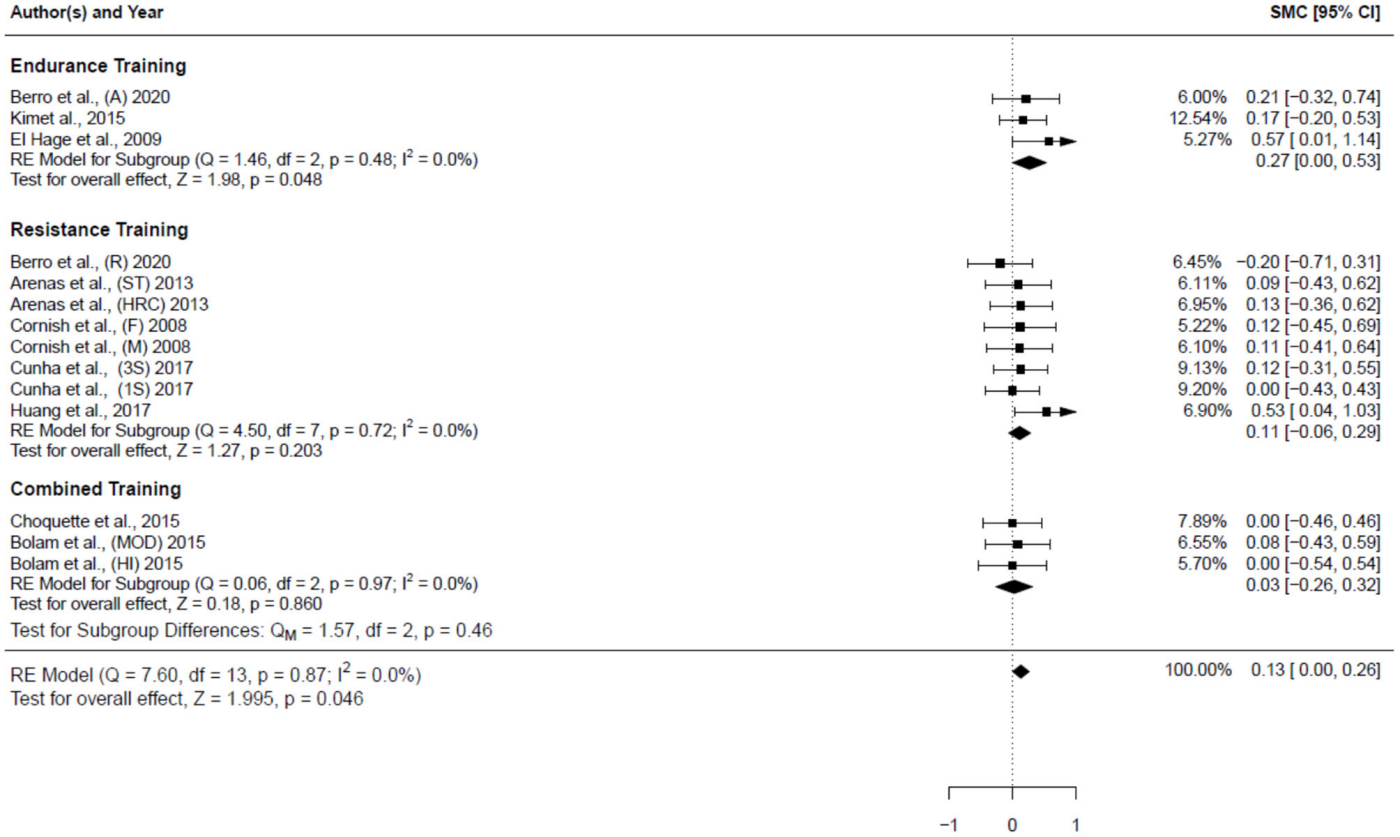
Forest plot for the estimated standardized effect size changes in WB BMD. Three subgroups were included endurance training, resistance training, and combined training. The black squares represent the standardized mean difference, while the left and right extremes of the squares represent the corresponding 95% confidence intervals. The middle of the black diamond represents the overall standardized mean difference, while the left and right extremes of the diamond represent the corresponding 95% confidence intervals. SMC, standardized mean change; A, aerobic; R, resistance; ST, strength training; HRC, Hight resistance circuit; F, females; M, males; 3S, 3 sets; 1S, 1 set; MOD, moderate dose impact loading; HI, high-dose impact loading.

**Figure 3:**
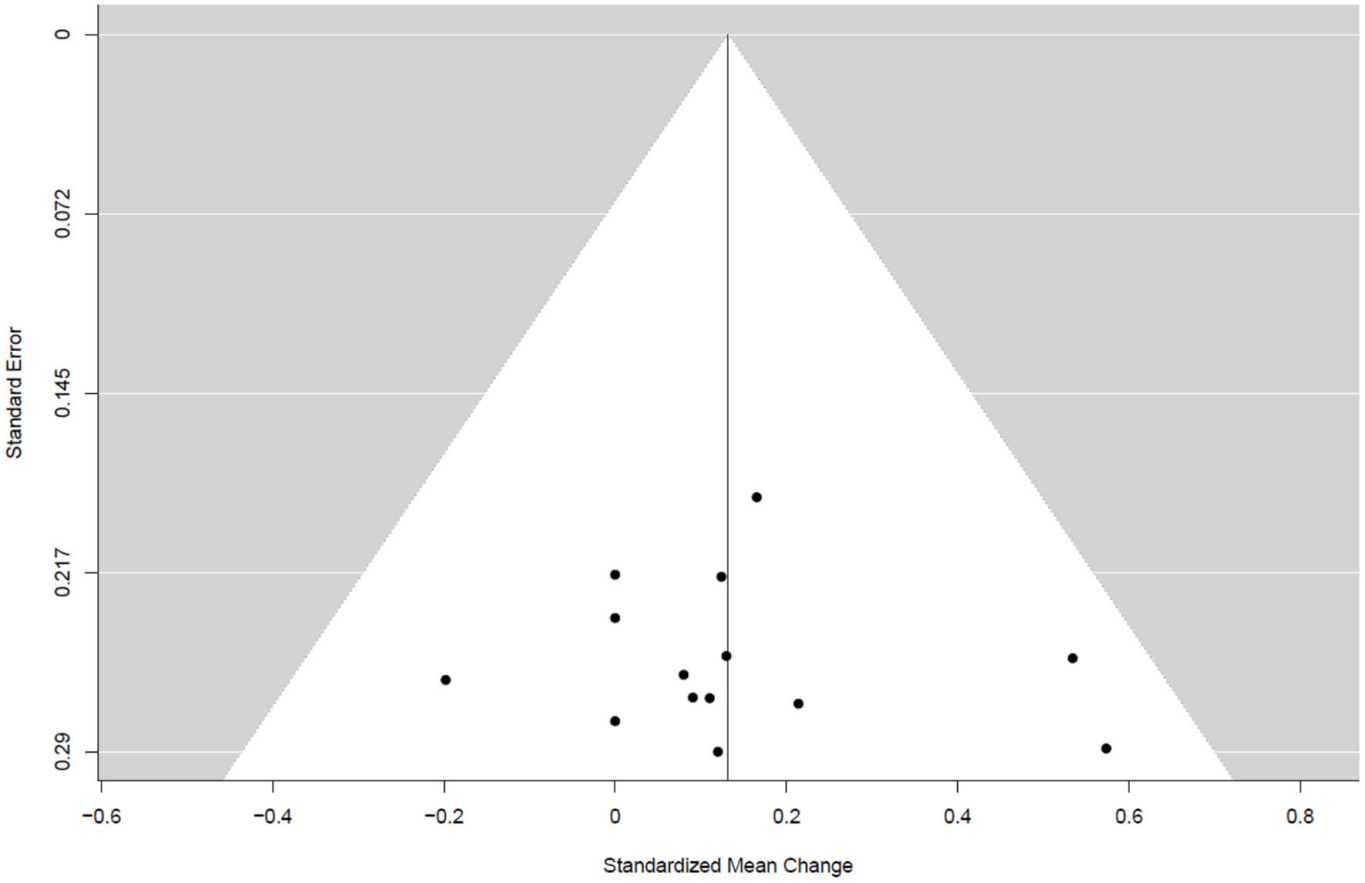
Funnel plots for WB BMD in the whole group.

Table 4 summarizes the studies that examined the effects of exercise training (endurance training, strength and resistance training, and combined training) on bone health indices in individuals with obesity. Nine studies, consisting of 14 trained groups (8 for females, 5 for men, and 1 for men and females) and 240 participants, were included in the meta-analysis investigating the effect of exercise on the WB BMD, as shown in the forest plot (Figure 2). Our analysis indicated a significant trivial increase in the WB BMD (0.13 SMD; 95% CI [0.00, 0.26], *p* = 0.046). The *I*^2^ (0.0%) parameter indicated the homogeneity of the results, whilst the visual inspection of the funnel plot (Figure 3) did not reveal a substantial asymmetry.

**Table 4:** Studies examined the effects of exercise training on bone health indices in individual with obesity.

	**Reference**	**Gender, Number of participants (N), and age (yrs)**	**Intervention**	**Outcomes 1 physical performance, weight, body composition**	**Outcomes 2 DMO, CMO, bone geometry, hormones**	**Effect size SMD [95% CI]**
Endurance training	El Hage et al., 2009	Women; N: 21 Ex1 normal Weight: 7 Ex2 overweight: 8 Ex3 obese: 6 Age: 16.2	Ex1, Ex2, and Ex3: 3d*w ×90–60 min per session, running at 70% MAV, strengthening and proprioceptive exercises, stretching, and collective games.	No modifications on weight and body composition.	Ex2 and Ex3: ↑ Legs BMC Ex3: ↑ WB BMC. Ex1, Ex2 and Ex3: ↑ total and sub-total BMD for the 3 groups.	WB BMD; 0.57 [0.01, 1.14]
	Kim et al., 2015	Men; N: 39 Ex: 29 CG: 10 Age: 25.3	CG: no exercise Ex: 4d*w, 65–75% VO_2_ max to burn ~600 Kcal per session.	Ex: ↓ weight, ↓ BMI, ↓ WC, ↓ trunk fat %, ↓ total fat %. CG: ↑ WC. Higher – weight changes, BMI, WC, trunk fat %, total fat % in Ex compared to CG.	Ex: no changes in BMD, ↓ FPI, ↓ HOMA-IR, ↑ HDL-C, ↓ LDL-C, total adiponectin ↓, ↓ leptin, ↑ HMW/TAdip, ↓ 1.25(OH)_2_D, ↑ OC, ↑_uc_OC, ↑ _uc_OC/OC. Higher – changes in FPI, HOMA-IR, LDL-C, total adiponectin, and leptin compared to CG. Higher + changes in HDL-C, HMW/TAdip, OC, and _uc_OC in Ex compared to CG.	WB BMD; 0.17 [−0.20, 0.53] Weight; −0.48 [−0.87, −0.10]
	Berro et al., 2020	Women; N: 28 Ex: 14 CG: 14 Age: 18–35	Ex: 3d*w, 45 min, 60% VO_2_ max, treadmill running CG: no exercise.	Ex: ↓ weight, ↓ BMI, ↓ FM, ↓ FM%, ↓ WC, ↓ HC, ↓ trunk FM%, ↑ maximal str, ↑ MAV. CG: ↑ weight, ↑ BMI, ↑ HC. Higher + changes in CSI, BSI, ISI, VJ, RMHS, MAV, and VO_2_ max (ml/mn/kg et l/mn) in the ETG compared to CG. Higher - weight changes, BMI, FM, FM%, WC, and HC in the Ex compared to CG.	Ex: ↑ WB BMC, ↑ L1-L4 BMD CG: ↓ CSI, ↓ BSI, ↓ ISI. Higher + changes in CSI, BSI, and ISI in the ETG compared to CG.	WBBMD; 0.21 [−0.32, 0.74] Weight; −0.32 [−0.85, 0.22]
Resistance or Strength training	Huang et al., 2017	Women; N: 35, Ex: 18 CG: 17 age >60 yrs	Ex: Elastic band resistance training (12 w, 3d*w), 55 min, 10 min of warm-up 40. min of elastic band RE and 5 min of cooling down. CG: a 40-min course about home exercise.	Ex: Fat in the right upper extremity, left upper extremity, total fat, and fat % had decreased. Higher – changes in the right upper extremity fat, total fat, and fat % in Ex compared to Con.	Ex: WB BMD ↑, Z-score and T-score ↑.	WB BMD; 0.53 [0.04, 1.03] Weight; −0.03 [−0.49, 0.43]
	Cunha et al., 2018	Women; N: 62 Ex1: 21 Ex2: 20 NCG: 21 Age: 68 yrs	3d*w, 12 w for Ex1 and Ex2. Ex1: 1 set of 10 to 15 reps per exercise. Ex2: 3 sets of 10 to 15 reps per exercise. CG: no exercise.	Ex1: ↑ Tstr, ↑ LM. Ex2: ↑ Tstr, ↑ LM, ↓ TBF%. Higher + change of Tstr in Ex2 compared to Ex1. Higher – changes of TBF% in Ex2 compared to Ex1.	No effect on bone density. Higher + changes of Z score in Ex2 compared to Ex1.	Ex1/WB BMD; 0.00 [−0.43, 0.43] Ex2/WB BMD; 0.12 [−0.31, 0.55]
	Cornish and Chilibeck, 2009	Women and men; N: 51 ALA: 25 ALA men: 14 ALA women: 11 Pla: 26 Pla men: 14 Pla women: 12 Age: 65.4	ALA G: Flaxseed oil (14g of ALA per day) + RT. Pla: 30ml of corn oil per day + RE. RE for both G: 3d*w progressive RE for major muscle groups.	ALA and Pla: ↑ leg press, ↑ chest press, ↑ LM, ↑ muscle thickness elbow flex and ext, ↑ knee ext, ↓ FM% and weight. Pla: women ↑ muscle knee flexor thickness. Higher + changes in muscle knee flexors thickness in ALA men. ALA men: ↓ IL-6 concentration.	ALA and Pla: ↑ hip BMC, ↑ hip BMD, ↑ WB BMC. Pla: WB BMD ↑.	Men/WBBMD; 0.11 [−0.41, 0.64] Women/WBBMD; 0.12 [−0.45, 0.69]
	Romero-Arenas et al., 2013	Women and men; N: 37 HRC: 16 ST: 14 CG: 7 Age: 61.6	ST: 2d*w, 2 sets of 3 exercises, 12 reps at 50% of 6RM, 10 reps at 75% of 6RM, 1 min rest between exercises. HRC: the same exercise as ST, exercises executed consequently in 2 circuits separated with 5 min. CG: no exercise.	HRC and ST: ↑ isokinetic str, ↑ LM Higher + changes in isokinetic str in HRC and ST comp to CG. HRC: ↓ FM%, ↓ FM. Higher - changes in FM and FM% in HRC comp to CG. Higher + changes in LM in HRC comp to CG. HRC: ↑ VO_2LM_ (ml/kg*min) for 1, 2 and 3 min. HRC: ↓ energy expenditure for 1, 2, and 3 min.	HRC and ST: ↑ WB BMD.	HRC/WB BMD; 0.13 [−0.36, 0.62] ST/WB BMD; 0.09 [−0.43, 0.62]
	Berro et al., 2020	Women; N: 29 Ex: 15 CG: 14 Age: 18–35	Ex: 3d*w, 45 min, 75% RM, 8 to12 reps, 4 to 5 exercises per muscle group. CG: no exercise.	Ex: ↓ weight, ↓ BMI, ↓ FM, ↓ FM%, ↓ WC, ↓ HC, ↑ LM, ↑ maximal str, ↑ MAV, TBS cor + %Δ VJ, TBS cor + %Δ RMHS. Higher + changes in the VJ, RMHS, MAV, and the VO_2_ max (ml/min/kg) in the Ex. Higher - changes in the weight, BMI, FM, FM%, WC, and HC in the Ex. CG: ↑ weight, ↑ BMI, ↑ HC, ↓ CSI, ↓ BSI, ↓ ISI.	Ex: ↑ WB BMC, ↑ L1-L4 BMD, ↑ TBS, ↑ SI, ↑ CSI, ↑ BSI, ↑ ISI. CG: ↑ WB ↑ BMI, ↓ CSI, ↓ BSI, ↓ ISI. Higher + changes in the L1-L4 BMD, CSI, BSI, ISI, in the Ex compared to CG.	WBBMD; −0.20 [−0.71, 0.31] Weight; −0.28 [−0.80, 0.23]
	Bocalini et al., 2009	Women; N: 35 Ex: 23 CG: 12 Age: 57–75	Ex: 3d*w, 10 min warm up, progressive RM (50% - 85%), 3 sets*10 reps for upper and lower muscles. CG: no exercise.	Ex: ↑ muscle str for the lower and upper body, ↓ weight. Higher – changes in the weight in the Ex compared to CG.	Ex: no changes in bone parameters. CG: ↓ LS BMD, ↓ FN BMD.	Weight; −0.19 [−0.61, 0.22]
Combined training	Bolam et al., 2016	Men; N: 42 HI: 13 Mod: 15 CG: 14 Age: 50–74 yrs	4 d*w, for 36 w. Upper body RE and either high-dose impact loading (HI; 80 jumps per session) or moderate-dose impact loading (MOD; 40 jumps per session).	Higher + changes in Arm LM in HI compared to CG. Higher + changes in 6-meter fast walk-in Mod compared to HI and CG.	Higher - changes in Hip BMD in the Mod G compared to HI and Con. Higher – changes in Troc BMD in Mod G compared to HI. No effects on Testosterone, SHBG, Estradiol.	HI/WB BMD; 0.00 [−0.54, 0.54] MOD/WB BMD; 0.08 [−0.43, 0.59]
	Choquette et al., 2011	Women; N: 100 -Pla, n: 26 -ISO, n: 26 -Ex + Pla, n: 25 -Ex + ISO, n: 23 Age: 50–70 yrs	Pla: Cellulose ISO: 70 mg daily dose of isoflavones. Ex + Pla: 1h*3d*w, 30 min of RT, and 30 min of ET. RE: free weights and selective plate machines, 1 set/12–14 reps/60% in M1 to 4sets/4–6 reps/85% in M6. ET: cycle ergometer and a treadmill, started at 40–50% of HR and increased up to 70–85%. -Ex + ISO.	Pla: leg FM ↓. ISO: leg FM ↓. Ex + Pla: ↓ HC, ↓ WC, ↓ TFM, ↓ Arms FM, ↓ Legs FM, ↑ TLM, ↑ Arms LM, ↑ Appendicular LM, ↓ %TFM, ↓ % Trunk FM, ↓ % Arms FM, ↓ % Legs FM. Ex + ISO: ↓ HC, ↓ WC, ↓ TFM, ↓ Arm FM, ↓ Legs FM, ↓ Trunk FM, ↑ TLM, ↑ Arms LM, ↑ Legs LM, ↑ Appendicular LM, ↓ %TFM, ↓ % Trunk FM, ↓ % Arms FM, ↓ % Legs FM.	ISO: ↓ TH BMD. Ex + Pla: ↑ LDL-C, ↑ Glucose. Pla: ↑ LDL-C. ISO: ↓ Insulin, ↓ Homa.	WB BMD; 0.00 [−0.46, 0.46] Weight; −0.06 [−0.52, 0.40]

*N, number of subject; G, group; C, control; Ex, exercise; d, day; W, week; reps, repetitions; RM, maximal resistance; VO_2_ max, maximal oxygen consumption; MAV, maximal aerobic velocity; HR, heart rate; ET, endurance training; RE, resistance exercise; HI, high-dose impact loading; MOD, moderate dose impact loading; BMC, bone mineral content; BMD, bone mineral density; WB, whole body; LS, lumbar spine; TH, total hip; FN, femoral neck; TBS, trabecular bone score; WC, waist circumference; HC, hip circumference; troc, trochanter; str, strength; Mstr, muscle strength; Tstr, total strength; FM, fat mass; TBF, total body fat; TFM, total fat mass; LM, lean mass; TLM, total lean mass; VJ, vertical jump; RMHS, maximal resistance half squat; CSI, compression strength index; BSI, binding strength index; ISI, impact strength index; SI, strength index; Alpha linolenic acid, ALA; ISO, isoflavones; Placebo, Pla; HOMA, homeostasis assessment model; %Δ, percentage of variation; FPI, fasting plasma insulin; HOMA-IR, homeostatic model assessment for insulin resistance; HDL-C, high-density lipoprotein cholesterol; LDL-C, low-density lipoprotein cholesterol; HMW/TAdip, ratio of high molecular weight adiponectin to total adiponectin; 1.25(OH)_2_D, 1,25-dihydroxy-Vitamin D; OC, osteocalcin; _uc_OC, undercarboxylated osteocalcin; IL-6, interleukin 6; ↑, increase; ↓, decrease*.

The subgroup of endurance training, as shown in the forest plot (Figure 2), included 3 studies (3 groups: 2 females and 1 man) and 57 participants. A significant small increase in the WB BMD (0.27 SMD; 95% CI [0.00, 0.53], *p* = 0.048) was detected among these studies, and the *I*^2^ (0.0%) parameter indicated homogeneity of the results.

The subgroup of resistance training (Figure 2) included 5 studies (8 groups: 6 females, 1 males, and 1 man and females) and 153 participants. The result showed a non-significant trivial increase in the WB BMD (0.11 SMD; 95% CI [−0.06, 0.29], *p* = 0.203) was detected among these studies. The *I*^2^ (0.0%) parameter indicated homogeneity of the results.

The subgroup of combined training (Figure 2) included 2 studies (3 groups: 1 females, 2 males) and involved 53 participants; it assessed the impact of the combined training on the WB BMD. A non-significant trivial increase in the WB BMD (0.03 SMD; 95% CI [−0.26, 0.32], *p* = 0.86) was detected among these studies. The *I*^2^ (0.0%) parameter indicates the homogeneity of the results. Concerning the impact of the type of physical training on the WB BMD, the comparison between the subgroups (Figure 2) did not reveal any significant differences (*p* = 0.46).

**Figure 4:**
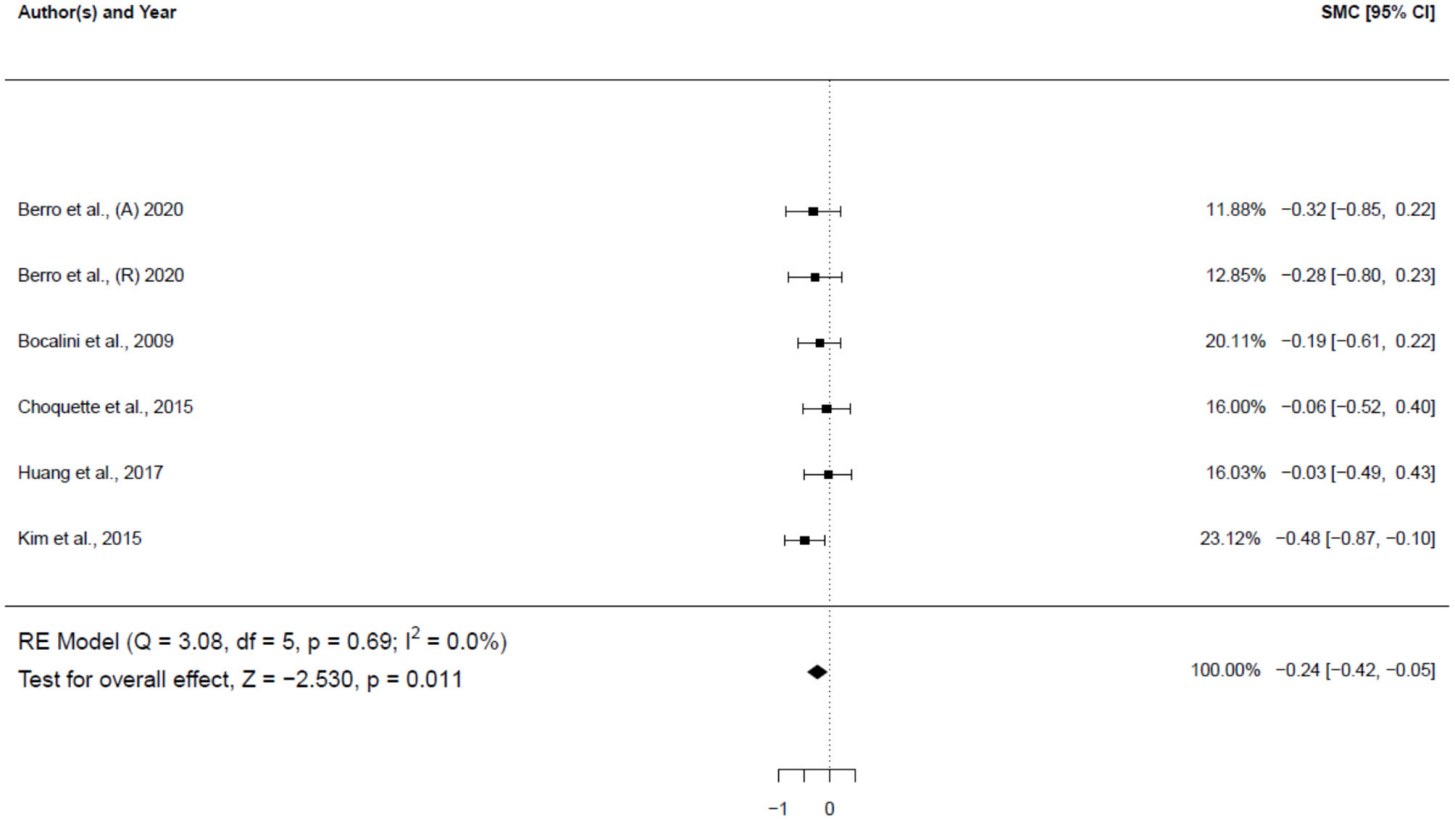
Forest plot for the estimated standardized effect size changes in weight. The black squares represent the standardized mean difference, while the left and right extremes of the squares represent the corresponding 95% confidence intervals. The middle of the black diamond represents the overall standardized mean difference, while the left and right extremes of the diamond represent the corresponding 95% confidence intervals. SMC, standardized mean change A, aerobic; R, resistance.

Five studies (6 groups: 5 females, 1 male), involving 124 participants, investigated the impact of physical activity on weight (Figure 4). The results showed a small significant decrease in the weight of subjects (−0.24 SMD; 95% CI [−0.42, −0.05], *p* = 0.011). The *I*^2^ (0.0%) parameter indicated homogeneity of the results.

## Discussion

To the best of our knowledge, this is the first meta-analysis to have investigated the influence of the type of physical activity intervention on bone mass in individuals with overweight/obesity. The results show that physical activity is likely to increase the WB BMD in individuals with obesity.

The findings of the present study are supportive of two recent meta-analyses (Ashe et al., 2021; Clemente et al., 2021). Ashe and colleagues (Ashe et al., 2021) suggested that physical activity has little effect on the total hip BMD, and little to no effect on femoral neck BMD, lumbar spine BMD, and WB BMD in males. The other meta-analysis assessed the effects of small-sided games-based training programs on BMD in untrained adults (Clemente et al., 2021) and reported a trivial effect and small effect for this type of training on the WB BMD and lower limbs BMD, respectively when compared to the control groups. The modest osteogenic expression found in the WB BMD, compared to other bone sites, could reflect a site-specific effect for physical exercise. In addition, while the benefits of weight-bearing exercises on bone are well established in the literature (Weaver et al., 2016; Beck et al., 2017), such benefits are generally related to baseline BMD. Accordingly, BMD is, therefore, not expected to largely increase in subjects with overweight, since they already have high BMD values (Qiao et al., 2020).

To further understand the influence of physical exercise on bone, it is important to better comprehend how varying weight statuses may be related to bone health. Accordingly, our results demonstrated a small, but significant, reduction in body weight. Indeed, this outcome extends previous meta-analytic results showing that physical activity promotes weight loss in adolescents with obesity (Stoner et al., 2019). Weight loss is generally associated with bone loss and increased fracture risk in both males and females (Cummings and Nevitt, 1994; Meyer et al., 1995, 1998; Langlois et al., 1996; Lee et al., 2010). Hence, this meta-analysis suggests that the osteogenic effect of the exercise could be tempered by the associated weight loss and that physical activity has the potential to overcome its detrimental effect on BMD among individuals with overweight/obesity. Indeed, these results support the beneficial use of a physical activity to promote weight loss and preserve bone mass in subjects with overweight/obesity.

### Effects of Exercise Training on Bone Health Indices in Individuals With Obesity

#### Effects of Endurance Training

Our analysis showed that endurance training slightly increases the bone density in individuals with obesity. The three studies included (El Hage et al., 2009; Kim et al., 2015; Berro et al., 2020) were of high quality, included both sexes, and the population was restricted to adolescents (El Hage et al., 2009) and adults (Kim et al., 2015; Berro et al., 2020). Two studies used treadmill running as an intervention (Kim et al., 2015; Berro et al., 2020), and the third one used team sports (El Hage et al., 2009). Albeit in the short duration period, the highest increase in the WB BMD was noted among adolescents with obesity who used collective aerobic exercise (El Hage et al., 2009) compared to the treadmill running adult populations (Kim et al., 2015; Berro et al., 2020). It is relatively well established in the literature that the potential of the osteogenic response during adolescence is higher compared to adulthood (Bonjour et al., 2009; Golden and Abrams, 2014). Interestingly, the osteogenic effect of the collective aerobic exercise appeared to be higher than the classic treadmill running. Aerobic exercise is a low-cost and easy to perform physical activity; indeed, these two characteristics are important when compliance is needed to obtain the desired effect. The studies included in this review indicated that weight-bearing aerobic exercise can have a positive impact on bone health among adolescents and adults with obesity.

#### Effects of Resistance or Strength Training

Our analysis demonstrated that resistance training has little to no effect on the WB BMD. The training programs ranged between 12 to 96 weeks and involved free weights, elastic bands, machines, and circuit training as the interventions for both genders. A previous meta-analysis (Kelley et al., 2001) examined the impact of the resistance exercise on BMD in females (consisting of 29 studies) and found that resistance exercise had a positive effect on BMD at the lumbar spine of all females, and the femur and radius sites for postmenopausal females. However, we did not include these parameters in our analytical model, which precludes any direct comparison.

In our analysis, seven papers had an average participant age of more than 55 years (Bocalini et al., 2009; Cornish and Chilibeck, 2009; Romero-Arenas et al., 2013; Huang et al., 2017; Cunha et al., 2018), and postmenopausal females represented the majority of the subjects. Age is an important factor which influences the responsiveness of bone to exercise. It was previously reported (Berger et al., 2010) that the peak bone mass (PBM) for the trabecular and cortical bone occurs before 40 and 33 years for females and males, respectively. Bone loss begins after PBM and accelerates in females with the onset of menopause (Weaver et al., 2016). Accordingly, physical activity must overcome bone loss before inducing any gain. In addition, as mentioned earlier, obesity is associated with higher BMD (Qiao et al., 2020); thus, the osteogenic response would be expected to be low among subjects with overweight/obesity.

The greatest increase in WB BMD was reported in the study of (Huang et al., 2017) despite the short duration of their training program. One possible explanation for this result is the trivial weight loss observed in this investigation. In fact, this study reported the smallest effect concerning weight loss among all studies. Also, these authors did not control for dietary factors, which can influence the impact of exercise, weight variation, and, consequently, the bone mass (Huang et al., 2017). Resistance training is recognized as an efficacious, non-pharmacological intervention to enhance bone mass (Weaver et al., 2016; Beck et al., 2017). Despite the absence of a significant effect, considering the population age and weight status, this result might suggest that the resistance exercise can combat the bone loss associated with aging in individuals with overweight/obesity.

#### Effects of Combined Training

This meta-analysis showed that the combined exercise training had little to no effect on the WB BMD. Two studies were included in the analysis; Choquette et al. (2011) and Bolam et al. (2016) investigated the influence of the combined exercise training on WB BMD in males and females with overweight/obesity, with an age range between 50 and 74 years. A previous meta-analysis in postmenopausal females showed that the risk of fractures in the combined exercise groups was significantly lower than in controls. The percentage change of BMD of the spine, trochanter, femoral neck, but not the total hip, was in favor of the intervention group. Our observed effect size may be attributable to the age and the weight status of participants, which can potentially diminish the osteogenic response of the physical exercise. Nevertheless, the present results suggest that the combined training may have the potential to combat bone loss in older adults.

Notably, the subgroups comparison did not reveal any significant differences among the types of training. The highest effect was observed in the endurance training, in which the age of the samples was lower compared to the other subgroups.

Although we have presented a novel addition to the literature, this meta-analysis has some limitations that must be mentioned. The number of studies that investigated the impact of endurance and combined training on bone health in subjects with overweight/obesity was relatively small. Furthermore, the training program's duration was highly variable, ranging from eight (Kim et al., 2015) to ninety-six (Warren and Schmitz, 2009) weeks. To this point, the resorption activity in a basic multicellular unit in adult human bone takes ~3 weeks and the formation response 3 to 4 months (Sims and Martin, 2014), which means that a longer program could induce a higher osteogenic effect and as such be more impactful. Also, age and gender are two well-known moderators of the relation between fat and bone (Dolan et al., 2017). For example, younger individuals tend to exhibit a higher osteogenic effect with physical activity compared to older subjects (Bonjour et al., 2009; Golden and Abrams, 2014). The studies incorporated in this analysis included both sexes, adolescents, adults, and the elderly. Unfortunately, due to the few number of studies we identified in our analysis, sex and age could not be considered as moderators, in addition to the type of physical exercise.

## Conclusion

Our systematic review and meta-analysis suggests that physical training have little to no effect on the WB BMD in subjects with overweight obesity. This conclusion, however, is based upon a limited number of available studies. Furthermore, there is also insufficient evidence at this time to advocate a specific type of exercise for enhancing bone health. Additional well-designed randomized controlled trials, investigating the impact of different types of physical exercise on bone health, in both sexes, and among individuals with overweight/obesity, especially during adolescence and adulthood, are needed before any firm recommendations can be made.

## Data Availability Statement

The original contributions presented in the study are included in the article/supplementary files, further inquiries can be directed to the corresponding author/s.

## Author Contributions

HZ, AJB, and RE contributed to the conception or designof the work. AJB, SK, AJ, and RE independently performed searches in the electronic databases, evaluated articles, and extracted data for the review. All authors confirm responsibility for the following: review conception and design, interpretation reviewed the results, and approved the final version of results, manuscript preparation, writing, editing, the manuscript.

## Conflict of Interest

The authors declare that the research was conducted in the absence of any commercial or financial relationships that could be construed as a potential conflict of interest.

## Publisher's Note

All claims expressed in this article are solely those of the authors and do not necessarily represent those of their affiliated organizations, or those of the publisher, the editors and the reviewers. Any product that may be evaluated in this article, or claim that may be made by its manufacturer, is not guaranteed or endorsed by the publisher.
